# Aspectos metodológicos do estudo *Comercialização de Alimentos em Escolas Brasileiras*

**DOI:** 10.1590/0102-311XPT167624

**Published:** 2025-05-23

**Authors:** Raquel Canuto, Sabrina Gomes Ferreira Clark, Luiza Delazari Borges, Paulo César Pereira de Castro, Letícia Ferreira Tavares, Letícia de Oliveira Cardoso, Ariene Silva do Carmo, Larissa Loures Mendes

**Affiliations:** 1 Universidade Federal do Rio Grande do Sul, Porto Alegre, Brasil.; 2 Universidade Federal de Pernambuco, Recife, Brasil.; 3 Universidade Federal de Minas Gerais, Belo Horizonte, Brasil.; 4 Instituto de Nutrição, Universidade do Estado do Rio de Janeiro, Rio de Janeiro, Brasil.; 5 Instituto de Nutrição Josué de Castro, Universidade Federal do Rio de Janeiro, Rio de Janeiro, Brasil.; 6 Escola Nacional de Saúde Pública Sergio Arouca, Fundação Oswaldo Cruz, Rio de Janeiro, Brasil.

**Keywords:** Estudantes, Espaço Social Alimentar, Estudos Transversais, Students, Food Social Space, Cross-sectional Studies, Estudiantes, Espacio Social y Comida, Estudios Transversales

## Abstract

Este artigo apresenta os aspectos metodológicos da concepção e organização do estudo *Comercialização de Alimentos em Escolas Brasileiras*, que teve como objetivo avaliar aspectos relacionados à comercialização de alimentos e bebidas em escolas privadas, a partir de informações coletadas com gestores de cantinas escolares e ambulantes presentes no entorno imediato das escolas. Trata-se de um estudo transversal em cantinas escolares e ambulantes no entorno de escolas privadas de Ensinos Fundamental e Médio nas 26 capitais brasileiras. Foi empregada amostragem aleatória simples com reposição inversa, ao final, foram avaliadas 2.241 cantinas e 699 ambulantes. A proporção de perdas amostrais foi de 21,2%. O questionário foi desenvolvido e avaliado por especialistas contemplando questões sobre: informação alimentar e nutricional, infraestrutura para alimentação, conveniência, preço, disponibilidade, variedade e promoção. A logística contou com uma equipe coordenadora que conduziu os treinamentos, realizou a gestão e controle da qualidade dos dados, pesquisadores locais e uma empresa que auxiliaram no trabalho de campo. As coletas ocorreram entre 2022 e 2024. As principais dificuldades de implementação foram sua concomitância com o período eleitoral de 2022, episódios de violência nas escolas, políticas internas das escolas e a complexidade da diversidade cultural e a vasta extensão territorial brasileira. Como pontos fortes destacam-se o ineditismo dos dados coletados em profundidade e representatividade, o uso das evidências geradas para proposição de políticas públicas de regulação do ambiente alimentar escolar e o apoio na construção de uma rede de pesquisadores na temática.

## Abstract (en)

This study presents the methodological aspects of the design and organization of the *Food Commercialization in Brazilian Schools* study, which aimed to evaluate aspects related to the commercialization of food and beverages in private schools, based on information collected from managers of school cafeterias and street vendors in the immediate vicinity of schools. This is a cross-sectional study of school cafeterias and street vendors near private primary and secondary schools in the 26 Brazilian state capitals. Simple random sampling with inverse replacement was employed; ultimately, 2,241 cafeterias and 699 street vendors were evaluated. The sample loss rate was 21.2%. The questionnaire was developed and evaluated by experts and included questions on nutritional and food information, food infrastructure, convenience, price, availability, variety, and promotion. The logistics involved a coordinating team that conducted training, managed and controlled data quality, local researchers, and a company that assisted in fieldwork. Data collection was conducted from 2022 to 2024. The main implementation challenges were its concurrence with the 2022 electoral period, episodes of violence in schools, internal school policies, the complexity of cultural diversity, and the vast territorial extension of Brazil. Strengths include the novelty of the in-depth and representative data collected, the use of the generated evidence to propose public policies for regulating the school food environment, and the support in building a network of researchers in this area.

## Resumen

Este estudio presenta los aspectos metodológicos de diseño y organización de la investigación *Comercialización de Alimentos en Escuelas Brasileñas*, que tuvo como objetivo evaluar los aspectos relacionados con la comercialización de alimentos y bebidas en escuelas privadas a partir de datos recopilados de los comedores escolares y de los vendedores ambulantes en las inmediaciones de las escuelas. Este es un estudio transversal realizado en comedores escolares y en los vendedores ambulantes en las inmediaciones de las escuelas primaria y secundaria privadas en 26 capitales brasileñas. Se utilizó muestreo aleatorio simple con reemplazo inverso; y al final se evaluaron 2.241 comedores y 699 vendedores ambulantes. La proporción de pérdidas de la muestra fue del 21,2%. El cuestionario fue desarrollado y evaluado por expertos y contenía preguntas sobre información alimentaria y nutricional, infraestructura alimentaria, conveniencia, precio, disponibilidad, variedad y promoción. La logística contó con un equipo que coordinó la capacitación, realizó la gestión y control de calidad de los datos, con investigadores locales y una empresa que asistió en el trabajo de campo. La recolección de datos tuvo lugar entre 2022 y 2024. Las principales dificultades de implementación fueron su concomitancia con el período electoral 2022, episodios de violencia en las escuelas, políticas internas de las escuelas y la complejidad de la diversidad cultural y la vasta extensión territorial brasileña. Los puntos fuertes incluyen la novedad de los datos recopilados en profundidad y representatividad, el uso de la evidencia generada para proponer políticas públicas para regular el entorno alimentario escolar y el apoyo en la formación de una red de investigadores sobre el tema.

## Introdução

O ambiente em que o indivíduo está inserido apresenta importante influência nos hábitos alimentares desde a infância ^1^ ^,^ ^2^, sendo um cenário com potencial para ações de prevenção da saúde no nível populacional ^3^ ^,^ ^4^. Estudos já demonstraram que a comercialização de alimentos não saudáveis dentro da escola está associada ao maior consumo desses alimentos entre os alunos ^5^ ^,^ ^6^. Uma parte significativa do consumo de alimentos não saudáveis é atribuída à comercialização desses nas cantinas escolares e/ou dos pontos de venda de alimentos dentro ou a uma curta distância das escolas ^7^ ^,^ ^8^. 

A *Pesquisa Nacional de Saúde do Escolar* de 2019 (PeNSE 2019) revelou que 31,4% dos estudantes de escolas públicas têm acesso a cantinas e 54,8% têm acesso a ponto alternativo de venda de alimentos e/ou bebidas no entorno das escolas, como comércio ambulante, onde são oferecidos diversos alimentos não saudáveis. Em comparação, na rede privada, 88% dos estudantes têm acesso a cantinas, e 72,6% dos estudantes consomem alimentos comercializados nelas ^9^. Além disso, a análise de dados do *Estudo de Riscos Cardiovasculares em Adolescentes* (ERICA) de 2013 e 2014 mostrou que as cantinas de escolas particulares apresentaram com maior frequência propaganda e venda de alimentos ultraprocessados, em comparação às públicas ^10^. 

Nesse sentido, estudar o ambiente alimentar escolar é fundamental para a compreensão dos fatores relacionados ao consumo alimentar de crianças e adolescentes. No Brasil, poucos estudos investigaram de maneira específica a comercialização dos alimentos e bebidas no ambiente alimentar escolar ^11^ ^,^ ^12^, considerando aspectos como o preço dos alimentos e o seu propósito e o grau de processamento dos alimentos, o número de opções de alimentos nos cardápios ^13^, as estratégias de comunicação mercadológica ^14^ e a infraestrutura das cantinas ^15^, principalmente, no contexto das escolas privadas. Por isso, o estudo *Comercialização de Alimentos em Escolas Brasileiras* (Caeb) foi concebido com o propósito de investigar de forma ampla aspectos fundamentais do ambiente alimentar escolar de escolas privadas brasileiras. Optou-se por investigar o ambiente alimentar de escolas privadas com cantina de capitais brasileiras, porque as escolas públicas (i) são regulamentadas pelo Programa Nacional de Alimentação Escolar (PNAE), (ii) já existe um conjunto de evidências na literatura que avaliam a implementação do PNAE ^16^, e (iii) alguns estados, como Rio Grande do Sul ^17^ e Rio de Janeiro ^18^, possuem leis que proíbem a comercialização de alimentos e bebidas em escolas públicas. 

Nesse sentido, ao desvelar os aspectos do ambiente alimentar escolar das escolas privadas, pretende-se também fornecer subsídios para a implementação de legislações que regulem a distribuição, oferta, comercialização, propaganda e publicidade de alimentos e bebidas ultraprocessadas. Esses dispositivos legais contribuem para a construção de um ambiente escolar comprometido com o bem-estar das crianças e adolescentes ^19^ ^,^ ^20^.

O presente artigo apresenta e discute aspectos metodológicos da concepção e organização geral do estudo Caeb, desde a elaboração do desenho do estudo e plano amostral até a produção dos instrumentos de coleta de dados, atividades de treinamento e detalhamento da coleta de dados. Também são discutidos os desafios para a condução de um estudo de base escolar nas capitais brasileiras, com o intuito de auxiliar outros pesquisadores no planejamento de futuras pesquisas sobre o ambiente alimentar escolar.

## Materiais e métodos

### Desenho de estudo

Trata-se de um estudo transversal de base escolar que incluiu cantinas escolares e ambulantes no entorno de escolas privadas de Ensinos Fundamental e Médio nas 26 capitais brasileiras e no Distrito Federal. Realizado de 2022 a 2024, o objetivo principal foi avaliar aspectos relacionados à comercialização de alimentos e bebidas em escolas privadas, a partir de informações coletadas com gestores de cantinas escolares e ambulantes presentes no entorno imediato das escolas. Os aspectos avaliados foram a disponibilidade de alimentos, a qualidade nutricional, os preços praticados e as estratégias de comunicação mercadológica.

### Amostra e amostragem

#### Cantinas

Foram incluídas no estudo escolas privadas com Ensinos Fundamental e Médio das capitais brasileiras e do Distrito Federal que possuíssem cantina, definida como dependência dentro da escola destinada à comercialização de alimentos para alunos, professores e toda a comunidade escolar ^17^. As escolas que atendem apenas Ensino Infantil não foram incluídas na amostra por apresentarem diferentes modelos de comercialização e consumo de alimentos (kit lanche, lanches trazidos de casa, lanches coletivos) e, muitas vezes, não possuírem cantinas. Foram excluídas as escolas com Ensinos Fundamental e Médio, com ≤ 50 alunos matriculados ^21^ ^,^ ^22^.

Para definição da amostra, partiu-se das informações do catálogo de escolas do Instituto Nacional de Estudos e Pesquisas Educacionais Anísio Teixeira (INEP) de 2021 ^23^. As informações utilizadas das escolas foram: nome, código INEP, endereço, município, Unidade Federativa, telefone, dependência administrativa e etapas e modalidades de ensino oferecidas. Porém, a unidade de análise do estudo foram as cantinas. Essa informação não está disponível no catálogo. Por isso, para estimar o percentual de escolas privadas de Ensinos Fundamental e Médio que possuíam cantina, foi utilizada uma prevalência estimada de 60% das escolas privadas localizadas nas capitais brasileiras, descrita no trabalho de Souza et al. ^24^ a partir dos dados da PeNSE 2015. Uma triagem telefônica nas cidades de Recife (Pernambuco), Porto Alegre (Rio Grande do Sul) e Belo Horizonte (Minas Gerais) verificou a presença de cantinas nas escolas listadas no catálogo, confirmando a estimativa de que aproximadamente 60% das escolas possuíam cantina. 

Dessa forma, o cálculo amostral para cada cidade considerou como universo a presença de cantinas em 60% das escolas e foi definido para que fosse possível obter estimativas de prevalência dos desfechos investigados de em torno de 50%, com erro amostral de 5% e um nível de 95% de confiança, a partir da fórmula abaixo:

$$n=Ν.{Ζ}^{2}.p.\left(1-p\right)/{Ζ}^{2}.p.\left(1-p\right)+e^{2}.Ν-1$$

Onde: n = tamanho da amostra obtido por meio do cálculo; N = população finita; Z = desvio indicado ao valor médio aceitável para que o nível de confiança seja atingido (sendo utilizado valor de z = 1,96, uma vez que o nível de significância é de 95%); p = real probabilidade do evento (utilizou-se o valor de 0,5, considerando a prevalência de 50%); e = margem de erro amostral (utilizou-se o valor de 0,05, considerando a margem de erro de 5%).

Optou-se por uma amostragem aleatória simples, com método de amostragem inversa, para garantir a aleatoriedade da reposição ao longo da coleta de dados ^25^ ^,^ ^26^. Nas cidades que por questões logísticas e financeiras foi viável, todas as escolas elegíveis foram convidadas a participar. Na Tabela 1, é possível observar os parâmetros amostrais, bem como a amostra final para cada uma das cidades.

**Tabela 1:** Parâmetros amostrais do estudo *Comercialização de Alimentos em Escolas Brasileiras* (Caeb).

Capital (Estado)	População finita *	População finita corrigida **	Tamanho amostral estimado
Aracaju (SE)	131	78	65
Belém (PA)	231	138	102
Belo Horizonte (MG)	348	208	135
Boa Vista (RR)	30	18	17
Campo Grande (MS)	116	71	60
Cuiabá (MT)	94	56	49
Curitiba (PR)	173	104	82
Distrito Federal	330	198	131
Florianópolis (SC)	71	42	38
Fortaleza (CE)	546	327	177
Goiânia (GO)	263	157	112
João Pessoa (PB)	159	93	75
Macapá (AP)	39	23	22
Maceió (AL)	218	131	98
Manaus (AM)	192	115	89
Natal (RN)	172	103	81
Palmas (TO)	37	22	21
Porto Alegre (RS)	97	58	51
Porto Velho (RO)	40	24	23
Recife (PE)	385	231	144
Rio Branco (AC)	26	16	15
Rio de Janeiro (RJ)	1.016	609	236
Salvador (BA)	635	380	191
São Luís (MA)	288	172	119
São Paulo (SP)	1.294	776	257
Teresina (PI)	126	75	63
Vitória (ES)	34	20	19
Total	7.091	4.245	2.472

AC: Acre; AL: Alagoas; AM: Amazonas; AP: Amapá; BA: Bahia; CE: Ceará; ES: Espírito Santo; GO: Goiás; MA: Manaus; MG: Minas Gerais; MS: Mato Grosso do Sul; MT: Mato Grosso; PA: Pará; PB: Paraíba; PE: Pernambuco; PI: Piauí; PR: Paraná; RJ: Rio de Janeiro; RR: Roraima; RN: Rio Grande do Norte; RO: Rondônia; RS: Rio Grande do Sul; SC: Santa Catarina; SE: Sergipe; SP: São Paulo; TO: Tocantins.

* População finita de escolas segundo catálogo de escolas do Instituto Nacional de Estudos e Pesquisas Educacionais Anísio Teixeira (INEP) ^23^;

** Correção da população finita de escolas (segundo base da INEP), considerando que 60% dessas escolas possuem cantinas, sendo elegíveis para o estudo.

A partir da listagem inicial das escolas (INEP), os pesquisadores locais realizaram uma triagem para verificar se a escola estava em funcionamento e se havia a presença de cantina (critérios de inclusão). A triagem foi realizada por meio de contato telefônico, site institucional e redes sociais. Os contatos por e-mail e telefone foram sempre a primeira opção; delimitaram-se cinco tentativas de comunicação por e-mail e cinco tentativas de ligações em dias e horários (manhã ou tarde) alternados para cada escola. Foram consideradas inelegíveis as escolas com as quais não foi possível estabelecer contato. Nas escolas em que havia mais de uma cantina, todas as cantinas foram avaliadas.

#### Ambulantes

A análise do comércio ambulante no entorno das escolas teve como objetivo contemplar o componente do entorno físico informal do ambiente alimentar escolar. O comércio ambulante de alimentos consiste na venda de alimentos e bebidas prontos para consumo imediato ou posterior, sem necessidade de preparo adicional, realizada por profissionais autônomos e informais que atuam em vias públicas, com horário e local flexíveis ^27^ ^,^ ^28^. Como o comércio ambulante geralmente não possui registro formal em órgãos da administração pública, o que impede o conhecimento preciso do número total de ambulantes, foi mantido o cálculo amostral utilizado para as cantinas. 

Foram considerados elegíveis os ambulantes que comercializavam alimentos e bebidas prontos para consumo no entorno imediato das escolas - definido como a porta das escolas, todas as calçadas imediatamente adjacentes à entrada/saída e nas calçadas laterais das unidades educacionais, bem como no quarteirão imediato, desde que o ponto de venda estivesse voltado para a portaria de entrada/saída da escola. Dessa forma, foram abordados os ambulantes presentes ocasionalmente no entorno imediato de cada escola participante, no momento da visita agendada para a entrevista com o cantineiro. Nas escolas em que havia mais de um ambulante, todos foram convidados a participar da pesquisa.

### Elaboração do questionário e variáveis

Os instrumentos que serviram de base para a elaboração dos instrumentos dessa pesquisa foram: instrumento de avaliação do ambiente alimentar *-* ESAO-r restaurantes) ^29^, o instrumento de auditoria do ambiente alimentar baseado na Nova Classificação de Alimentos do Guia Alimentar (Nova) - Audit-Nova ^30^, o instrumento de avaliação do ambiente alimentar universitário ^31^, o instrumento da *Pesquisa Nacional de Saúde do Escolar* (PeNSE) ^32^ e o instrumento do Projeto Segurança Alimentar em Cantinas Escolares dos Municípios do Território da Cidadania do Noroeste Colonial/RS ^33^. Na elaboração dos itens, foi garantida a inclusão de alimentos com base nas recomendações do *Guia Alimentar para a População Brasileira* ^1^ e no instrumento de avaliação do ambiente alimentar universitário ^31^, considerando aspectos da cultura alimentar regional. 

Os instrumentos passaram por avaliação de especialistas, tendo em conta três aspectos sobre os itens propostos: sua relevância, considerando os objetivos do projeto; a clareza da redação dos itens e das opções de respostas, levando-se em conta a ausência de ambiguidade. A confiabilidade foi verificada em uma amostra de conveniência de escolas públicas e privadas de uma capital e sua região metropolitana, por meio dos testes interobservador e intraobservador.

A versão final dos instrumentos desenvolvidos para avaliação das cantinas e ambulantes abrangeu as dimensões: informação alimentar e nutricional, infraestrutura para alimentação, conveniência, acessibilidade financeira, disponibilidade, variedade e promoção. Contemplaram questões distribuídas em três seções: (i) informações da cantina ou do ambulante (identificação e caracterização); (ii) comercialização, variedade, tamanho, valor do menor preço e estratégia de venda (promoção/combo) dos alimentos, bebidas e preparações culinárias; e (iii) presença de publicidade dos alimentos e bebidas (Quadro 1). Os instrumentos completos encontram-se disponíveis no site do estudo: https://estudocaeb.nutricao.ufrj.br/.

**Quadro 1:** Conteúdo e dimensões avaliadas nas seções dos instrumentos do estudo *Comercialização de Alimentos em Escolas Brasileiras* (Caeb).

SEÇÕES	CONTEÚDO	DIMENSÕES AVALIADAS	REFERÊNCIAS [AUTOR (ANO)]
1ª seção: identificação e caracterização da cantina e identificação e caracterização do vendedor ambulante	Aspectos administrativos (tipo de administração, número de clientes e de funcionários, forma de pagamento) *	Conveniência	Franco et al. ^31^ (2022); Balestrin et al. ^33^ (2022); Caspi et al. ^46^ (2012)
	Aspectos sociodemográficos, socioeconômicos e ocupacionais do vendedor ambulante (gênero, idade, escolaridade, cor/raça, principal fonte de renda, tempo de trabalho como ambulante, motivos por ter optado pelo trabalho) **	Socioeconômica	Monteiro ^47^ (2015)
	Aspectos estruturais, logísticos do comércio ambulante (localização, tipo e estrutura do local do comércio **	Conveniência e infraestrutura para alimentação	Ambikapathi et al. ^28^ (2021); Castro & Canella ^48^ (2022); Caspi et al. ^46^ (2012)
	Aspectos funcionais, operacionais e regulatórios do comércio ambulante, (comercialização de alimentos/bebidas em outro local, período de funcionamento, impedimento/proibição da atividade na porta das escolas e interferência da gestão escolar nos itens comercializados) **	Conveniência, política	Caspi et al. ^46^ (2012)
	Presença do nutricionista *	Governança	Balestrin et al. ^33^ (2022); Castro & Canella ^48^ (2022)
	Tipo de refeições ofertadas *	Governança	Balestrin et al. ^33^ (2022); Castro & Canella ^48^ (2022)
	Local onde as preparações culinárias comercializadas são produzidas * **	Infraestrutura para a produção de refeições	Balestrin et al. ^33^ (2022)
	Disponibilização de cardápios *	Disponibilidade	Franco et al. ^31^ (2021); Caspi et al. ^46^ (2012)
	Informações alimentares e nutricionais das preparações culinárias que compõem o cardápio disponíveis para os clientes *	Informações sobre alimentação e nutrição	Duran et al. ^29^ (2015); Franco et al. ^31^ (2021); Castro & Canella ^48^ (2022)
	Presença estrutura como mesa/balcão para consumo de lanche ou refeição no local *	Infraestrutura para alimentação	Franco et al. ^31^ (2021); Castro & Canella ^48^ (2022)
2ª seção: alimentos comercializados	Comercialização de alimentos para fins especiais *	Governança, política	Colares et al. ^49^ (2020)
	Critérios de seleção dos alimentos comercializados *	Governança	Balestrin et al. ^33^ (2022); Castro & Canella ^48^ (2022)
	Alimentos que são proibidos de serem comercializados *	Governança	Castro & Canella ^48^ (2022)
	Recebimento de algum material de incentivo/patrocínio/apoio de fornecedores dos produtos que comercializa. Em caso positivo, quais são os materiais e as empresas que patrocinam * **	Promoção	Organização Mundial da Saúde ^50^ (2012)
	Ações que incentivam a alimentação saudável e presença de materiais educativos sobre alimentação saudável *	Governança, política	Castro & Canella ^48^ (2022)
	Preço da refeição *	Acessibilidade financeira	Franco et al. ^31^ (2021); Caspi et al. ^46^ (2012)
	Estratégia de venda (promoção/combo) *	Promoção	Duran et al. ^29^ (2015); Castro & Canella ^48^ (2022); Organização Mundial da Saúde ^50^ (2012)
	Venda de alimentos e bebidas por outras pessoas da comunidade escolar *		Balestrin et al. ^33^ (2022)
	Comercialização dos alimentos, bebidas e preparações culinárias	Disponibilidade	Ministério da Saúde ^1^ (2014); Instituto Brasileiro de Geografia e Estatística ^9^ (2021); Duran et al. ^29^ (2015); Borges & Jaime ^30^ (2019); Franco et al. ^31^ (2021); Balestrin et al. ^33^ (2022); Caspi et al. ^46^ (2012); Castro & Canella ^48^ (2022)
	Variedade dos alimentos, bebidas e preparações culinárias comercializados	Variedade	Ministério da Saúde ^1^ (2014); Duran et al. ^29^ (2015); Borges & Jaime ^30^ (2019); Franco et al. ^31^ (2021); Caspi et al. ^46^ (2012)
	Menor preço dos alimentos, bebidas e preparações culinárias comercializados	Acessibilidade financeira	Ministério da Saúde ^1^ (2014); Duran et al. ^29^ (2015); Borges & Jaime ^30^ (2019); Franco et al. ^31^ (2021); Caspi et al. ^46^ (2012); Castro & Canella ^48^ (2022)
	Estratégia de venda (promoção/combo) dos alimentos, bebidas e preparações culinárias comercializados	Promoção	Franco et al. ^31^ (2021); Castro & Canella ^48^ (2022); Organização Mundial da Saúde ^50^ (2012)
3ª seção: publicidade dos alimentos comercializados	*Banner*/cartaz do fornecedor * **	Promoção	Franco et al. ^31^ (2021); Castro & Canella ^48^ (2022); Organização Mundial da Saúde ^50^ (2012)
	*Banner*/cartaz do estabelecimento * **		
	Vestimenta * **		
	Réplica do produto *		
	Cardápio * **		
	Embalagem * **		
	Painel/televisão * **		
	*Folder* * **		
	*Displays* * **		
	Brindes * **		
	Aplicativo da escola/cantina *		

* Instrumento da cantina;

** Instrumento do comércio ambulante.

### Estudo pré-teste do instrumento e piloto com instrumentos e procedimentos

O pré-teste dos instrumentos foi realizado em uma escola privada de Belo Horizonte, por meio do instrumento impresso, e conduzido por uma pesquisadora treinada. Posteriormente, o estudo piloto com os instrumentos e procedimentos foi realizado em 56 cantinas de 54 escolas privadas de Niterói (Rio de Janeiro). Toda a logística foi testada no estudo piloto. Os resultados obtidos foram utilizados para refinar os instrumentos e os fluxos dos procedimentos.

### Logística do estudo

Todo o trabalho de campo foi realizado por equipes da Vox Populi, uma empresa com experiência em pesquisas no território nacional, com supervisão da comissão coordenadora da pesquisa. A comissão coordenadora da pesquisa foi composta por cinco docentes pesquisadores com base no Nordeste (Universidade Federal de Pernambuco), Sudeste (Universidade Federal de Minas Gerais, Universidade Federal do Rio de Janeiro e Fundação Oswaldo Cruz) e Sul (Universidade Federal do Rio Grande do Sul) e duas estudantes de doutorado.

### Estratégias de divulgação do estudo

A equipe coordenadora do estudo implementou estratégias para divulgá-lo e sensibilizar a comunidade escolar e, com isso, aumentar a adesão das escolas participantes. Entre as ações realizadas, destacam-se a criação de um site (https://estudocaeb.nutricao.ufrj.br/), que forneceu informações detalhadas sobre sua relevância, objetivos, metodologia, equipe de pesquisadores e financiadores. Adicionalmente, foram realizadas entrevistas com a imprensa, além de participação em audiências públicas do poder legislativo municipal, onde os pesquisadores puderam compartilhar os principais aspectos do estudo.

### Pesquisadores locais

Foi estabelecida parceria com pesquisadores de 23 instituições de Ensino Superior públicas brasileiras (a composição da equipe está disponível no site do estudo). Os pesquisadores colaboradores locais auxiliaram na operacionalização do estudo, desde a tramitação nos comitês de ética das instituições, passando pelo auxílio nas triagens telefônicas, até o acompanhamento do trabalho de campo.

### Treinamento dos entrevistadores

Todos os pesquisadores parceiros envolvidos e entrevistadores foram treinados e instruídos sobre o processo de coleta dos dados. Foi desenvolvido um manual instrutivo que auxiliou na padronização da coleta dos dados. Também foi criado um treinamento em vídeo, com duração de 40 minutos, reproduzido para as equipes de cada cidade participante, seguido por uma simulação prática da coleta, com duração de duas horas, para familiarizar os entrevistadores com o sistema do formulário digital e aspectos estratégicos do contato, agendamento e condução das auditorias e entrevistas.

Os treinamentos ocorreram em modalidade remota, sempre que uma nova equipe local fosse formada para o início do período de coleta de dados, e foram conduzidos por duas doutorandas membros da coordenação do estudo.

### Coleta dos dados

O contato telefônico com as escolas elegíveis para o agendamento da coleta de dados foi realizado por entrevistadores recrutados pela empresa. Durante o contato telefônico com a escola, com o objetivo de localizar o(a) gestor(a) da cantina, foi feita a apresentação da pesquisa e a solicitação de agendamento da visita à cantina. Além disso, cabia aos entrevistadores a verificação dos endereços das escolas e o planejamento das rotas de visitas em seu território, o agendamento, o registro de aceite do termo de consentimento e a coleta de dados.

Quanto aos ambulantes, os entrevistadores percorreram o entorno das escolas participantes, identificando ambulantes elegíveis e convidando-os para participar do estudo.

As coletas ocorreram de junho de 2022 a junho de 2024, em dias letivos, segundo o calendário escolar municipal, nos turnos da manhã ou tarde. Nas cantinas, cada entrevista durou, em média, 30 minutos. Com ambulantes, o tempo variou entre 10 e 15 minutos.

### Gestão dos dados e controle de qualidade

O treinamento e capacitação da equipe de campo, a coleta digital georreferenciada, a checagem das entrevistas e a análise da consistência dos dados foram as ações desenvolvidas para monitorar, identificar e corrigir potenciais riscos à qualidade dos dados.

A coleta foi realizada de forma totalmente digital. A transmissão dos dados criptografados, bem como das coordenadas geográficas do local da entrevista, ocorreu em tempo real a partir do dispositivo eletrônico (celular ou *tablet*) utilizado pelo entrevistador, com a instalação de um aplicativo do sistema CollectBox (https://www.collectbox.com.br/) conectado à internet.

Posteriormente, os dados foram exportados para uma planilha no Microsoft 365 Excel (https://products.office.com/) para checagem. A checagem consistiu na realização do retorno ao campo em uma proporção de 20% das entrevistas realizadas, sendo observada a concordância com os dados coletados, o que dispensou a necessidade de implementação de outras medidas. Essa etapa foi conduzida por uma equipe específica da empresa contratada, distinta da equipe responsável pela coleta inicial.

Complementarmente, foi mantido o acompanhamento remoto ao longo do estudo. Semanalmente, foram compartilhadas atualizações sobre os agendamentos de visitas, bem como sobre a geração de dados coletados. Também foram compartilhados os dados de geolocalização do entrevistador, com o intuito de garantir que estes correspondessem ao endereço da escola selecionada para o estudo. Quinzenalmente, foram realizadas reuniões entre a coordenação do estudo e a empresa responsável pela equipe de coleta para discutir o perfil de desenvolvimento da coleta em cada cidade (ritmo, pontos facilitadores e resistências). Mensalmente, ocorreram reuniões entre a coordenação do estudo, pesquisadores locais e a empresa para apresentação do cenário resumido do trabalho de campo (número de entrevistas, agendamentos, demandas de contato entre o pesquisador local e a escola para elucidação de dúvidas, número de perdas, ajustes de calendário e previsão de conclusão da coleta) e para o enfrentamento de dificuldades, além de ajustes nos protocolos conforme particularidades locais.

### Questões éticas

O estudo seguiu os preceitos éticos descritos na *Declaração de Helsinki* e nas *Resoluções nº 466/2012* e *nº 510/2016*, do Conselho Nacional de Saúde. Obteve-se aprovação pelos Comitês de Ética em Pesquisa e Seres Humanos das universidades públicas parceiras nas localidades de execução do estudo (Material Suplementar; https://cadernos.ensp.fiocruz.br/static//arquivo/supl-e00167624_4530.pdf). Foram incluídos no estudo os(as) gestores(as) de cantina e os vendedores ambulantes que concordaram em participar e registraram o aceite no Termo de Consentimento Livre e Esclarecido (TCLE). Os participantes também foram consultados sobre a permissão para registro fotográfico do ponto de comércio. Ressalta-se que a coleta de dados respeitou os Princípios de Privacidade da Lei Geral de Proteção de Dados Pessoais (LGPD).

## Resultados

A coleta de dados ocorreu em 26 capitais brasileiras e no Distrito Federal. A triagem de elegibilidade envolveu 7.576 contatos com escolas privadas cadastradas no INEP, das quais 3.021 (39,9%) eram elegíveis, e destas, 2.180 (72,2%) aceitaram participar. Foram avaliadas 2.241 cantinas que funcionavam em escolas privadas e 699 ambulantes no entorno imediato das escolas.

O estudo apresentou uma proporção de perdas de 13,5%. Considerando a amostra teórica baseada na presença de cantinas em 60% das escolas privadas nas capitais brasileiras, a proporção de perdas foi de 12,5%.

A Tabela 2 reúne dados da triagem de elegibilidade até o número final de cantinas avaliadas, de escolas participantes e a proporção de perdas para cada cidade. A amostra pretendida foi alcançada em nove cidades. Apenas em quatro cidades o número de escolas com cantina revelou-se inferior à projeção teórica da amostra, após verificação por triagem telefônica, sendo elas Campo Grande (Mato Grosso do Sul), Fortaleza (Ceará), Porto Velho (Rondônia) e Salvador (Bahia) (Tabela 2). Este cenário impossibilitava o alcance da amostra pretendida, que considerava a presença de cantinas em 60% das escolas, mesmo se não houvesse qualquer perda e/ou recusa nessas quatro cidades. O número de entrevistas realizadas com ambulantes, por cidade, também está disponível na Tabela 2.

**Tabela 2:** Amostra final do estudo *Comercialização de Alimentos em Escolas Brasileiras* (Caeb): amostragem aleatória simples.

Capital (Estado)	Tamanho amostral estimado	Escolas elegíveis	Escolas convidadas a participar	Escolas participantes	Proporção de perdas (%) *	Cantinas avaliadas **	Ambulantes avaliados ***
Aracaju (SE)	65	73	73	71	2,7 ^#^	71	16
Belém (PA)	102	200	102	97	4,9	103	25
Belo Horizonte (MG)	135	148	135	74	45,2	76	4
Boa Vista (RR)	17	17	17	16	5,9	16	5
Campo Grande (MS)	60	59	59	53	10,2 ^#^	58	17
Cuiabá (MT)	49	53	53	50	5,7 ^#^	53	0
Curitiba (PR)	82	104	82	79	3,7	81	99
Distrito Federal	131	218	218	177	18,8 ^#^	179	58
Florianópolis (SC)	38	40	40	39	2,5 ^#^	39	0
Fortaleza (CE)	177	142	142	106	25,3 ^#^	113	0
Goiânia (GO)	112	183	114	114	0,0	115	0
João Pessoa (PB)	75	77	75	63	16,0	65	2
Macapá (AP)	22	30	30	29	3,3 ^#^	29	4
Maceió (AL)	98	103	98	90	8,2	96	97
Manaus (AM)	89	89	89	61	31,5	62	41
Natal (RN)	81	82	81	72	11,1	75	9
Palmas (TO)	21	34	21	21	0,0	21	19
Porto Alegre (RS)	51	67	67	60	10,4 ^#^	60	11
Porto Velho (RO)	23	16	16	16	0,0 ^#^	17	2
Recife (PE)	144	160	144	120	16,7	124	69
Rio Branco (AC)	15	20	20	20	0,0 ^#^	21	2
Rio de Janeiro (RJ)	236	256	236	194	17,8	200	106
Salvador (BA)	191	145	145	126	13,1 ^#^	126	25
São Luís (MA)	119	124	119	107	10,1	108	25
São Paulo (SP)	257	464	261	261	0,0	262	51
Teresina (PI)	63	93	63	51	19,0	56	12
Vitória (ES)	19	24	19	13	31,6	15	0
Total	2.077	3.021	2.519	2.180	13,5	2.241	699

AC: Acre; AL: Alagoas; AM: Amazonas; AP: Amapá; BA: Bahia; CE: Ceará; ES: Espírito Santo; GO: Goiás; MA: Manaus; MG: Minas Gerais; MS: Mato Grosso do Sul; MT: Mato Grosso; PA: Pará; PB: Paraíba; PE: Pernambuco; PI: Piauí; PR: Paraná; RJ: Rio de Janeiro; RR: Roraima; RN: Rio Grande do Norte; RO: Rondônia; RS: Rio Grande do Sul; SC: Santa Catarina; SE: Sergipe; SP: São Paulo; TO: Tocantins.

* Proporção de perdas considerando as escolas elegíveis convidadas a participar do estudo;

** Nas escolas participantes em que havia mais de uma cantina, todas foram avaliadas;

*** Número total de ambulantes participantes considerando todas as escolas visitadas. Em algumas escolas não havia ambulantes; em outras, havia mais de um, e todos foram convidados a participar;

^#^ Cidades onde todas as escolas elegíveis foram convidadas a participar, mesmo após a amostra ter sido alcançada.

Quanto aos ambulantes, nas cidades de Cuiabá (Mato Grosso), Fortaleza, Florianópolis (Santa Catarina), Goiânia (Goiás) e Vitória (Espírito Santo) não foram encontrados ambulantes. Como os ambulantes foram abordados antes ou depois das entrevistas nas cantinas escolares, isso pode não ter coincido com os horários de maior movimento de estudantes, como na entrada ou saída dos turnos escolares.

Os resultados da análise da comercialização de alimentos e bebidas nas escolas privadas geraram um resumo executivo sobre a situação geral de cada município. Esses resumos foram disponibilizados no site da pesquisa para consulta dos participantes e gestores escolares, conforme divulgação antecipada no momento do convite à participação. Os resultados municipais, juntamente com o *Guia Prático para uma Cantina Saudável* ^34^, elaborado pela equipe do estudo Caeb, foram entregues em formato impresso no contexto de uma nova abordagem à cantina, no desenvolvimento de um estudo longitudinal correlato.

## Discussão

O estudo Caeb foi pioneiro ao coletar dados sobre a comercialização de alimentos e bebidas em escolas privadas de todas as capitais brasileiras, utilizando um método padronizado e especificamente desenvolvido para avaliar a comercialização de alimentos em cantinas e entre ambulantes. Anteriormente, este tema era abordado de forma secundária em inquéritos nacionais sobre a saúde de crianças e adolescentes ^9^ ^,^ ^35^. O desenvolvimento de dois instrumentos de coleta e manuais de implementação de cantinas saudáveis representa um avanço metodológico no estudo do ambiente alimentar escolar, que pode servir de base para futuras pesquisas.

Outro aspecto inovador do estudo foi a criação de um banco de dados com informações sobre cantineiros e ambulantes, algo que não estava disponível anteriormente, pois os órgãos públicos apenas possuíam informações cadastrais sobre as escolas, sem incluir os comerciantes de alimentos. Contudo, o estudo não cobriu a totalidade dos ambulantes atuantes no entorno das escolas participantes, dado que a abordagem aos ambulantes ocorreu apenas durante as entrevistas com os cantineiros, devido a limitações logísticas e orçamentárias. Isso restringiu a amostra de ambulantes à conveniência da coleta junto às cantinas.

O estabelecimento de contato com as escolas, como localização dos pontos de comercialização, demandou desde o esforço teórico do planejamento amostral até a verificação de informações viabilizada pela articulação com pesquisadores locais. A colaboração com pesquisadores locais foi crucial para o sucesso do estudo, pois eles possuíam um entendimento profundo da realidade e das sensibilidades culturais regionais, facilitando a comunicação com as escolas e aumentando a adesão ao projeto. Essa parceria também foi fundamental para garantir a precisão da coleta de dados, especialmente nas áreas de difícil acesso ou com particularidades socioculturais.

Outro aspecto importante deste estudo foi o seu potencial em gerar evidências para proposição de políticas públicas e incidência política em ações de *advocacy*, com consequência na diversidade de financiadores, atores públicos e do terceiro setor, e no uso dos resultados para tais ações na medida que eram produzidos. A proposição e aprovação de leis voltadas à proibição da comercialização de alimentos ultraprocessados em escolas nas cidades do Rio de Janeiro ^18^ e Niterói ^36^ foram embasadas em dados provenientes do estudo Caeb. Além disso, cumpre destacar que os dados do estudo têm sido utilizados pelos atores da sociedade civil que trabalham na agenda nas ações de incidência política em alguns estados e municípios brasileiros em parceria com o Fundo das Nações Unidas para a Infância (UNICEF) ^37^ ^,^ ^38^. Por fim, a perspectiva é de que os dados do estudo possam também fornecer subsídios para a implementação do *Decreto nº 11.821/2023* ^39^, que prevê que estados, Distrito Federal e municípios criem legislações que visem a regulamentar o ambiente escolar em consonância com os eixos estratégicos e as diretrizes para promover a alimentação adequada e saudável estabelecidos no decreto.

A contratação de uma empresa especializada em coleta de dados para recrutar e formar equipes de entrevistadores em cada cidade, prática usual em estudos no país ^40^ ^,^ ^41^, possibilitou a coleta simultânea de dados em diferentes regiões, sob a coordenação contínua de pesquisadores que orientavam a empresa. Durante esse processo, um fator importante para o sucesso do estudo foi assegurar a precisão da localização dos entrevistadores durante a gestão do trabalho de campo simultâneo, especialmente considerando a vasta e desafiadora extensão territorial do Brasil. Isso foi possível devido ao uso de dispositivos eletrônicos conectados à internet, que permitiram o georreferenciamento em tempo real das atividades dos entrevistadores, além de possibilitar a verificação do preenchimento completo do instrumento, a gravação e o armazenamento seguro dos dados da pesquisa. 

A condução deste trabalho também apresentou desafios. A expansão da abrangência territorial para todas as capitais ocorreu de forma gradual, viabilizada pela captação contínua de recursos financeiros, o que resultou em uma maior duração do estudo. Além disso, essa ampliação trouxe desafios relacionados à diversidade sociocultural dos estados, com dificuldades no trabalho de campo manifestando-se de maneira distinta. Entre os principais desafios enfrentados, destacam-se o período eleitoral de 2022, eventos violentos em escolas brasileiras, políticas internas das instituições de ensino e a grande extensão territorial. Esses fatores contribuíram para uma distribuição desigual da proporção de perdas entre as capitais.

A realização de uma pesquisa científica durante o período eleitoral brasileiro demandou o recrutamento de entrevistadores também envolvidos em pesquisas de intenção de voto, gerando uma disputa no mercado que desacelerou a coleta de dados entre agosto e setembro de 2022. No ano seguinte, os episódios de massacres em escolas resultaram em maior negativa ao acesso dos entrevistadores às escolas. Em meados de abril de 2023, a coleta de dados em Salvador precisou ser interrompida por vinte dias até que a percepção de segurança nas escolas aumentasse. 

Dificuldades também foram enfrentadas em relação à política interna de acesso às escolas. O discurso anticiência e a desvalorização dos pesquisadores e das universidades públicas propagado pela extrema direita brasileira, por meio de *fake news* que tentam minar a confiança dos brasileiros no método científico ^42^, foram fatores que dificultaram o acesso às escolas. Além disso, a coleta foi impactada pela resistência da população às medidas regulatórias propostas pelo Estado. Em Belo Horizonte, por exemplo, com a revogação do *Decreto nº 47.557/2018* ^43^ (que regulamentou a Lei da Cantina Saudável, *Lei nº 15.072/2004* ^44^), observou-se maior recusa por parte dos cantineiros, que estavam preocupados com possíveis fiscalizações e com a incerteza sobre a continuidade da vigência da legislação.

Em outro cenário, escolas de alto padrão econômico, principalmente em Vitória, Fortaleza e São Paulo, não permitiram a condução da pesquisa em suas cantinas, resultando em possível viés de seleção. Outras pesquisas apresentam a mesma dificuldade em obter informações de população de alta renda no Brasil ^45^. 

Por fim, a grande diversidade cultural e a vasta extensão territorial brasileira acrescentaram complexidade ao estudo devido ao difícil acesso terrestre em algumas cidades. Em Manaus (Amazonas), várias escolas cadastradas na base de dados do Inep estavam localizadas em áreas adjacentes de difícil acesso para os entrevistadores. Situação semelhante foi observada em Belém (Pará). Em Salvador, o crescimento desordenado da cidade gerou desafios adicionais, com a extensa área periférica, carente de cobertura de transporte público, limitando as opções de deslocamento dos entrevistadores.

Em suma, um estudo dessa magnitude enfrentaria desafios proporcionais à sua complexidade. O estudo Caeb, seguindo rigor metodológico, adaptou-se às condições da pesquisa de campo em escolas no período histórico em que foi realizado. Os resultados e discussões gerados só foram possíveis devido à criação de uma rede sólida de pesquisadores engajados no tema. 

Como proposições para pesquisas futuras no ambiente alimentar de escolas privadas, sugere-se: (1) a sensibilização da comunidade escolar, incluindo os pais, para importância desse tipo de estudo e de medidas regulatórias que protejam o ambiente escolar; (2) o estabelecimento de parcerias com pesquisadores e universidade locais que conheçam a realidade sociocultural do município e possam apoiar todo o trabalho de campo e também a análise e discussão dos achados; (3) o estabelecimento de parcerias com o terceiro setor que atua na agenda pública em alimentação e nutrição para que os resultados possam ser aplicados em ações de *advocacy*; (4) a contratação de empresa para coleta de dados como uma forma importante de viabilizar a pesquisa em todo território nacional; (5) a investigação indispensável do comércio informal do entorno da escola e que integre também o ambiente alimentar digital.

## Conclusão

Neste artigo, foi apresentada a metodologia empregada no estudo Caeb, suas potencialidades e limitações. Espera-se que essas informações possam auxiliar na elaboração do percurso metodológico de futuras pesquisas que tenham foco na compreensão do ambiente alimentar escolar no Brasil. Além disso, espera-se que seus resultados possam embasar a proposição de medidas regulatórias nas diferentes capitais brasileiras, ou até mesmo uma lei com abrangência nacional, visando incentivar escolhas alimentares saudáveis nas escolas. Isso contribuiria para o alcance do potencial máximo de crescimento e desenvolvimento das crianças brasileiras, bem como para a prevenção de todas as formas de má nutrição e de doenças crônicas não transmissíveis.

## References

[1] Ministério da Saúde (2014). Guia alimentar para a população brasileira..

[2] Swinburn BA, Sacks G, Hall KD, McPherson K, Finegood DT, Moodie ML (2011). The global obesity pandemic shaped by global drivers and local environments. Lancet.

[3] Yancey AK, Kumanyika SK, Ponce NA, McCarthy WJ, Fielding JE, Leslie JP (2003). Population based interventions engaging communities of color in healthy eating and active living a review. Prev Chronic Dis.

[4] Velásquez-Meléndez G, Mendes LL, Padez CMP (2013). Built environment and social environment associations with overweight and obesity in a sample of Brazilian adults. Cad Saúde Pública.

[5] Briefel RR, Crepinsek MK, Cabili C, Wilson A, Gleason PM (2009). School food environments and practices affect dietary behaviors of US public school children. J Acad Nutr Diet.

[6] Leite MA, Azeredo CM, Peres MFT, Escuder MML, Levy RB (2022). Availability and consumption of ultra-processed foods in schools in the municipality of São Paulo, Brazil results of the SP-Proso. Cad Saúde Pública.

[7] Monteiro CA, Levy RB, Claro RM, Castro IRR, Cannon G (2010). A new classification of foods based on the extent and purpose of their processing. Cad Saúde Pública.

[8] Leite FHM, Oliveira MA, Cremm EC, Abreu DSC, Maron LF, Martins PA (2012). Oferta de alimentos processados no entorno de escolas públicas em área urbana. J Pediatr (Rio J.).

[9] Instituto Brasileiro de Geografia e Estatística (2021). Pesquisa Nacional de Sau´de do Escolar: 2019.

[10] Carmo AS, Assis MM, Cunha CF, Oliveira TRPR, Mendes LL (2018). The food environment of Brazilian public and private schools. Cad Saúde Pública.

[11] Gabriel CG, Santos MV, Vasconcelos FAG, Milanez GHG, Hulse SB (2010). Cantinas escolares de Florianópolis existência e produtos comercializados após a instituição da lei de regulamentação. Rev Nutr.

[12] Willhelm FF, Ruiz E, Oliveira AB (2010). Cantina escolar qualidade nutricional e adequação à legislação vigente. Revista HCPA.

[13] Santos MF, Fagundes AA, Gabriel CG (2017). Caracterização das cantinas comerciais de escolas estaduais no município de Aracaju, Sergipe. Rev Baiana Saúde Pública.

[14] Gaetani RS, Ribeiro LC (2015). Produtos comercializados em cantinas escolares do município de Ribeirão Preto. Rev Bras Promoç Saúde.

[15] Porto EBS, Schmitz BAS, Recine E, Rodrigues MLCF (2015). School canteens in the Federal District, Brazil and the promotion of healthy eating. Rev Nutr.

[16] Canella DS, Bandeira L, Oliveira ML, Castro S, Pereira AS, Bandoni DH (2022). Update of the acquisition parameters of the Brazilian National School Feeding Program based on the Dietary Guidelines for the Brazilian Population. Cad Saúde Pública.

[17] Rio Grande do Sul (2020). Decreto nº 54.994, de 17 de janeiro de 2020. Regulamenta a Lei nº 15.216, de 30 de julho de 2018, que dispõe sobre a promoção da alimentação saudável e proíbe a comercialização de produtos que colaborem para a obesidade, diabetes e hipertensão em cantinas e similares instalados em escolas públicas e privadas do Estado do Rio Grande do Sul.. Diário Oficial do Estado.

[18] Rio de Janeiro (2023). Decreto Rio nº 52.842, de 11 de julho de 2023. Regulamenta a Lei Municipal nº 7.987, de 11 de julho de 2023, que institui ações de combate à obesidade infantil, e dá outras providências.. Diário Oficial da Prefeitura do Rio de Janeiro.

[19] Mahesh R, Vandevijvere S, Dominick C, Swinburn B (2018). Relative contributions of recommended food environment policies to improve population nutrition results from a Delphi study with international food policy experts. Public Health Nutr.

[20] São Paulo Portaria Conjunta COGSP/CEI/DSE, de 23 de março de 2005. Normas para funcionamento de cantinas escolares.. Diário Oficial do Estado de São Paulo.

[21] Bandoni DH, Ottoni IC, Amorim ALB, Canella DS (2024). Its time free meals at schools for all. Br J Nutr.

[22] Amorim ALB, Santos RD, Ribeiro JRS, Canella DS, Bandoni DH (2022). The contribution of school meals to food security among households with children and adolescents in Brazil. Nutrition.

[23] Instituto Nacional de Estudos e Pesquisas Educacionais Anísio Teixeira Microdados da educação básica 2021..

[24] Souza LBO, Azevedo ABC, Bandoni DH, Canella DS (2021). Characteristics of Brazilian school food and physical activity environments PeNSE 2015. Rev Saúde Pública.

[25] Haldane JBS (1945). On a method of estimating frequencies. Biometrika.

[26] Lima-Costa MF, Andrade FB, Souza PRB, Neri AL, Duarte YAO, Castro-Costa E (2018). The Brazilian Longitudinal Study of Aging (ELSI-Brazil): objectives and design.. Am J Epidemiol.

[27] World Health Organization (1996). Essential safety requirements for street-vended foods.

[28] Ambikapathi R, Shively G, Leyna G, Mosha D, Mangara A, Patil CL (2021). Informal food environment is associated with household vegetable purchase patterns and dietary intake in the DECIDE study empirical evidence from food vendor mapping in peri-urban Dar es Salaam, Tanzania. Glob Food Sec.

[29] Duran AC, Lock K, Latorre MR, Jaime PC (2015). Evaluating the use of in-store measures in retail food stores and restaurants in Brazil. Rev Saúde Pública.

[30] Borges CA, Jaime PC (2019). Development and evaluation of food environment audit instrument AUDITNOVA. Rev Saúde Pública.

[31] Franco JV, Garcia MT, Canella DS, Louzada IDR, Bógus CM (2021). Food environment at subway stations a study in the municipality of São Paulo, Brazil. Ciênc Saúde Colet.

[32] Instituto Brasileiro de Geografia e Estatística (2016). Pesquisa Nacional de Sau´de do Escolar: 2015.

[33] Balestrin M, Brasil CCB, Bellei EA, Marchi ACB, Kirsten VR, Wagner MB (2022). Cantinas Survey proposição e avaliação de um aplicativo para análise do risco sanitário e dos alimentos comercializados em cantinas escolares. RECIIS (Online).

[34] Vilela LA, Silva LEA (2023). Guia prático para uma cantina saudável.

[35] Okamura AB, Gonçalves VSS, Carvalho KMB (2022). School feeding as a protective factor against insulin resistance the Study of Cardiovascular Risks in Adolescents (ERICA). Int J Environ Res Public Health.

[36] Niterói (2023). Lei nº 3.766, de 5 de janeiro de 2023. Altera a Lei nº 2.659, de 19 de novembro de 2009, proíbe a comercialização, a aquisição, a confecção, a distribuição e a publicidade de produtos que contribuem para a obesidade infantil e dá outras providências.. Diário Oficial do Município de Niterói.

[37] Alimentando Políticas IDEC e UNICEF participam de agendas em Belém pelo avanço da promoção da alimentação escolar saudável..

[38] Instituto Brasileiro de Defesa do Consumidor IDEC e UNICEF se reúnem com governo da Bahia para promover alimentação adequada nas escolas..

[39] Brasil (2023). Decreto nº 11.821, de 12 de dezembro de 2023. Dispõe sobre os princípios, os objetivos, os eixos estratégicos e as diretrizes que orientam as ações de promoção da alimentação adequada e saudável no ambiente escolar.. Diário Oficial da União.

[40] Alves-Santos NH, Castro IRR, Anjos LA, Lacerda EMA, Normando P, Freitas MB (2021). General methodological aspects in the Brazilian National Survey on Child Nutrition (ENANI-2019) a population-based household survey. Cad Saúde Pública.

[41] Aquino EM, Barreto SM, Bensenor IM, Carvalho MS, Chor D, Duncan BB (2012). Brazilian Longitudinal Study of Adult Health (ELSA-Brasil) objectives and design. Am J Epidemiol.

[42] Silva AAM (2021). Aspectos metodológicos do Estudo Nacional de Alimentação e Nutrição Infantil (ENANI-2019). Cad Saúde Pública.

[43] Minas Gerais (2018). Decreto nº 47.557, de 10 de dezembro de 2018. Regulamenta a Lei nº 15.072, de 5 de abril de 2004, que dispõe sobre a promoção da educação alimentar e nutricional nas escolas públicas e privadas do sistema estadual de ensino.. Diário Oficial de Minas Gerais.

[44] Minas Gerais (2004). Lei nº 15.072, de 05 de abril de 2004. Dispõe sobre a promoção da educação alimentar e nutricional nas escolas públicas e privadas do sistema estadual de ensino.. Diário Oficial de Minas Gerais.

[45] Instituto Brasileiro de Geografia e Estatística (2023). Censo Demográfico 2022: população e domicílios. Primeiros resultados.

[46] Caspi CE, Sorensen G, Subramanian SV, Kawachi I (2012). The local food environment and diet a systematic review. Health Place.

[47] Monteiro MAM (2015). Caracterização do comércio ambulante de alimentos em Belo Horizonte-MG. Demetra (Rio J.).

[48] Castro IRR, Canella DS (2022). Organizational food environments advancing their conceptual model. Foods.

[49] Colares SS, Ferraz F, Perry ID, Soratto J (2020). Gestão do cuidado de estudantes com necessidades alimentares especiais vinculados ao Programa Nacional de Alimentação Escolar. Physis (Rio J.).

[50] World Health Organization (2012). A framework for implementing the set of recommendations on the marketing of foods and non-alcoholic beverages to children.

