# Supplementary material for: Methodological aspects of the study on Food Commercialization in Brazilian Schools
Source: Cad Saude Publica. 2025 May 23;41(5):e00167624. [Article in Portuguese] doi: 10.1590/0102-311XPT167624 (PMC12108110; doi:10.1590/0102-311XPT167624)
Supplement: Supplementary file 1 [file 1678-4464-csp-41-05-PT167624-s.pdf]

ASPECTOS ÉTICOS

Quadro 1 – Identificação e aprovação pelos respectivos comitês de ética.

Cidade/UF	CEP	CAAE	Parecer
Aracaju/SE	Universidade Federal de Sergipe	58002522.3.0000.5546	5.531.874
Belém/PA	Universidade Federal do Pará	61830322.3.0000.0018	5.913.835
Belo Horizonte/MG	Universidade Federal de Minas Gerais	38003220.4.0000.5149	5.240.459
Brasília/DF	Universidade de Brasília	57827222.7.0000.0030	5.427.066
Boa Vista/RR	Universidade Federal do Tocantins	70452923.0.1001.9187	6.942.713
Campo Grande/MS	Universidade Federal de Goiás	71101423.4.0000.5083	6.796.515
Cuiabá/MT	Universidade Federal do Mato Grosso	60895722.2.0000.8124	5.709.474
Curitiba/PR	Universidade Federal do Rio Grande do Sul	40784920.7.0000.5347	6.179.413
Florianópolis/SC	Universidade Federal de Santa Catarina	71142123.6.0000.0121	6.172.595
Fortaleza/CE	Universidade Federal de Pernambuco	56903722.0.0000.5208	6.002.739
Goiânia/GO	Universidade Federal de Goiás	71101423.4.0000.5083	6.270.770
João Pessoa/PB	Universidade Federal de Pernambuco	56903722.0.0000.5208	6.002.739
Macapá/AP	Universidade Federal do Tocantins	70452923.0.1001.9187	6.942.713
Maceió/AL	Universidade Federal de Pernambuco	56903722.0.0000.5208	6.002.739
Manaus/AM	Universidade Federal do Tocantins	70452923.0.1001.9187	6.942.713
Natal/RN	Universidade Federal de Pernambuco	56903722.0.0000.5208	6.002.739
Palmas/TO	Universidade Federal do Tocantins	70452923.0.1001.9187	6.942.713
Porto Alegre/RS	Universidade Federal do Rio Grande do Sul	40784920.7.0000.5347	4.506.207
Porto Velho/RO	Universidade Federal do Tocantins	70452923.0.1001.9187	6.942.713
Recife/PE	Universidade Federal de Pernambuco	56903722.0.0000.5208	5.446.216
Rio Branco/AC	Universidade Federal do Tocantins	70452923.0.1001.9187	6.942.713
Rio de Janeiro/RJ	UFRJ - Hospital Universitário Clementino Fraga Filho da Universidade Federal do Rio de Janeiro / HUCFF- UFRJ	44440820.4.0000.5257	5.060.837
Salvador/BA	Universidade Federal da Bahia	59777922.5.0000.5023	5.526.372
São Luís/MA	Universidade Federal de Pernambuco	56903722.0.0000.5208	6.002.739
São Paulo/SP	Universidade de São Paulo - Hospital das Clínicas da Faculdade de Medicina - HCFM/USP	67365822.5.0000.0068	5.959.036
Teresina/PI	Universidade Federal de Pernambuco	56903722.0.0000.5208	6.002.739
Vitória/ES	Universidade Federal do Espírito Santo	69976923.9.1001.5060	6.188.500

Fonte: Caeb, 2024.
